# Metabolic profiling of Alzheimer's disease brains

**DOI:** 10.1038/srep02364

**Published:** 2013-08-06

**Authors:** Koichi Inoue, Haruhito Tsutsui, Hiroyasu Akatsu, Yoshio Hashizume, Noriyuki Matsukawa, Takayuki Yamamoto, Toshimasa Toyo'oka

**Affiliations:** 1 Laboratory of Analytical and Bio-Analytical Chemistry, School of Pharmaceutical Sciences, University of Shizuoka, Shizuoka, Japan; 2 Department of Neuropathology, Choju Medical Institute, Fukushimura Hospital, Toyohashi, Japan; 3 Department of Neurology, Nagoya City University, Graduate School of Medical Sciences, Nagoya, Japan; 4 These authors contributed equally to this work.

## Abstract

Alzheimer's disease (AD) is an irreversible, progressive brain disease and can be definitively diagnosed after death through an examination of senile plaques and neurofibrillary tangles in several brain regions. It is to be expected that changes in the concentration and/or localization of low-molecular-weight molecules are linked to the pathological changes that occur in AD, and determining their identity would provide valuable information regarding AD processes. Here, we propose definitive brain metabolic profiling using ultra-performance liquid chromatography coupled with electrospray time-of-flight mass spectrometry analysis. The acquired data were subjected to principal components analysis to differentiate the frontal and parietal lobes of the AD/Control groups. Significant differences in the levels of spermine and spermidine were identified using S-plot, mass spectra, databases and standards. Based on the investigation of the polyamine metabolite pathway, these data establish that the downstream metabolites of ornithine are increased, potentially implicating ornithine decarboxylase activity in AD pathology.

Alzheimer's disease (AD) is a type of dementia that causes problems with memory, thinking and behavior among older people. In the AD brain, two abnormal structures called plaques and tangles have been the prime suspects in the severe depression of metabolic mechanisms, killing important nerve cells and impairing higher brain functions. Recently, various researchers compared a wide range of pathophysiological markers between mutation carriers and non-carriers as a function of the parental age at onset in order to evaluate the cascade of events such as clinical, cognitive, imaging, and biochemical measures in the large international cohort of AD patients^1^,^2^,^3^,^4^,^5^,^6^. In this result, the AD process begins more than 30 years before the clinical onset of dementia and is associated with a series of pathophysiological changes that occur over decades in cerebrospinal fluid (CSF) biomarkers related to plaques of amyloid beta (Aβ), tangles of tau protein, brain Aβ deposition and brain metabolism as well as progressive cognitive impairment^1^,^2^. Glucose metabolism in particular declines from the first stage of the disease to the onset of the expected symptoms (−30 years)^1^,^7^,^8^,^9^,^10^. Although we still do not know how the AD process begins and/or continues, it appears likely that a simple amyloid cascade hypothesis does not fit with the reality of the neurogenic etiology. Moreover, because the underlying cause of a diagnosis of dementia varies, it is not easy to identify the mechanism and brain regions involved in causing AD. So far as known, it is the key of AD process that this pathology and metabolic mechanisms may be contributed to the distinct cognitive profile in brain. Therefore, it is critical to identify the link between metabolic processes and the disease pathology that causes the distinct cognitive profile and brain lesions of AD patients. AD can be definitively diagnosed after death through definitive examination of senile plaques and fibrillary tangles in several brain regions using staining procedures such as Gallyas-Braak (GB). It would be very useful to be able to correlate metabolic changes in specific regions of the brain with the actual pathological changes that occur in AD. Monitoring various metabolites in the brain will enable the creation of multilateral framework to determine the pathophysiological features and processes of the neurogenic etiology.

Metabolic profiling of various AD patients using mass spectrometry (MS) techniques revealed that some low-molecular-weight molecules were decreased/increased in the AD versus control groups^11^. Recently, liquid chromatography coupled with mass spectrometry (LC/MS) lipidomic analysis indicated a fundamental change in the AD brain such that the lipid changes may contribute to pathogenesis^12^. Lipidomic MS used to identify lipid abnormalities showed that stearoyl-CoA desaturase and sphingolipids were elevated in the AD brain^13^,^14^. The greater sensitivity and definitive brain metabolic profiling by MS allows for the identification of a larger number of metabolites compared to other biological samples, which is especially useful for fingerprinting the pathogenesis of AD. However, the identification of biomarkers through less-invasive techniques can often be achieved through comparison to known metabolites and/or database matching metabolites from easy-to-acquire fluids (plasma and CSF) from AD patients without using genotype, pathology and/or diagnostic data^11^,^12^,^13^,^14^,^15^,^16^,^17^,^18^,^19^. The dramatic change in various metabolic mechanisms in the AD brain is poorly understood.

In this study, we propose to use novel and definitive brain metabolic profiling using ultra-performance liquid chromatography coupled with electrospray time-of-flight mass spectrometry (UPLC-ESI/TOF/MS) analysis to evaluate the diversity of low-molecular-weight molecules patterns from specific brain regions. The brain tissues used for metabolic profiling were therefore based on reliable backgrounds confirmed through multiple independent methods. Our study represents a major top-down use of definitive brain metabolomics using MS and multivariate data analysis regarding to AD pathology. We could identify specific metabolites such as spermidine (SPD) and spermine (SPM), and we discuss the metabolic mechanism of polyamine in specific brain region in the establishment of AD.

## Results

### Pathological examination of brain samples

**Figure 1:**
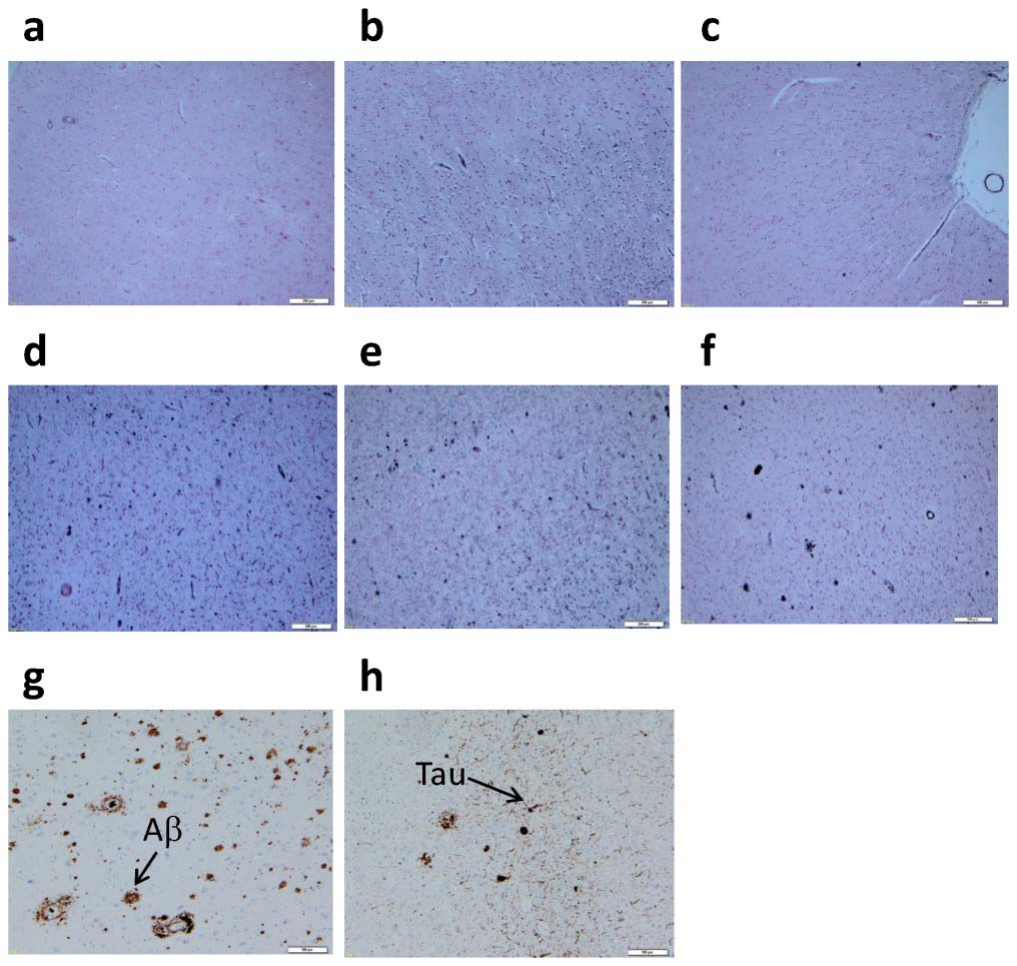
The plaque/tangle pattern of Gallyas-Braak (GB) and Aβ/Tau staining from representative AD/Control brain tissues. (a), Frontal lobe region from Control patient. (b), Parietal lobe region from Control patient. (c), Occipital lobe region from Control patient. (d), Frontal lobe region from AD patient. (e), Parietal lobe region from AD patient. (f), Occipital lobe region from AD patient. (g), Aβ staining from frontal lobe region (a). (h), Tau staining from frontal lobe region (a). For routine immunohistochemistry, an anti-human β amyloid antibody (Dako, mouse monoclonal, 1:30), an anti-human pTau antibody (INNOGENETICS, clone AT8 1:1000) were used for detection of plaque/tangle.

The descriptive diagnosis of all study variables is summarized in Table 1. In addition, the apolipoprotein-E (ApoE) genotype was 3/3 in all AD patients. In the AD group, we indicated the Braak Stage of AD in the post-mortem pathological diagnosis and the duration of disease ranged from 6 to 20 years pre-mortem. In the pathological examination of the AD brain, we assessed the functional properties of the visual pathology by determining the presence of plaques/tangles in brain tissues (Fig. 1). The plaques/tangles pattern of GB staining from AD/Control patients allowed each brain to be used in our comparison. Brain regions such as the frontal lobe (FL), parietal lobe (PL) and occipital lobe (OL) showed a pronounced propensity for GB staining properties in the AD/Control groups. Based on these results, we categorized the AD and Control groups for definitive brain metabolic profiles of low-molecular-weight molecules.

### UPLC-ESI/TOF/MS analysis of metabolites in brain tissues

We propose that MS metabolic profiling of AD pathology can be used to changes in brain metabolites related to the presence of plaques and tangles. Our previous protocol was applied for UPLC-ESI/TOF/MS data collection with a scan mode (positive/negative ionizations) from *m/z* 100–1000 using two different columns (T3-C18 and HS-F5) for pathologically confirmed AD/Control brain tissues^20^,^21^. Samples were prepared by centrifugal ultrafiltration to separate the low-molecular-weight phase for UPLC-ESI/TOF/MS profile analysis and to remove the protein phase (> 3,000 Da). Using these low-molecular-weight phases, the detected *m/z* values in MS chromatograms ranged from 1201 to 4699 compounds on positive and 385 to 1110 compounds on negative ionizations in all brain tissues (Fig. S1). These peak responses of low-molecular-weight molecules were extracted based on a 100 counts cutoff (marker intensity threshold) because of the dependability statistics. These raw data were analyzed for peak detection and alignment and then exported for a principal components analysis (PCA) and orthogonal partial least-squares-discriminant analysis (OPLS-DA).

For validation of UPLC-ESI/TOF/MS analysis, the pooled parietal lobe tissues from AD (n = 10) and Control (n = 10) were performed on the same day for determining the intra-day accuracy, replicate (n = 6) analytes of various peaks. The intra-day accuracy was expressed as the relative standard deviation (RSD, %) of retention time (0.08 – 1.63%) and peak area (1.44 – 8.93%) for typical *m/z* values (Table S1). For multivariate data analysis, each sample of real patients was used for single running based on this validation.

### Multivariate statistical analysis of metabolites in brain tissues

**Figure 2:**
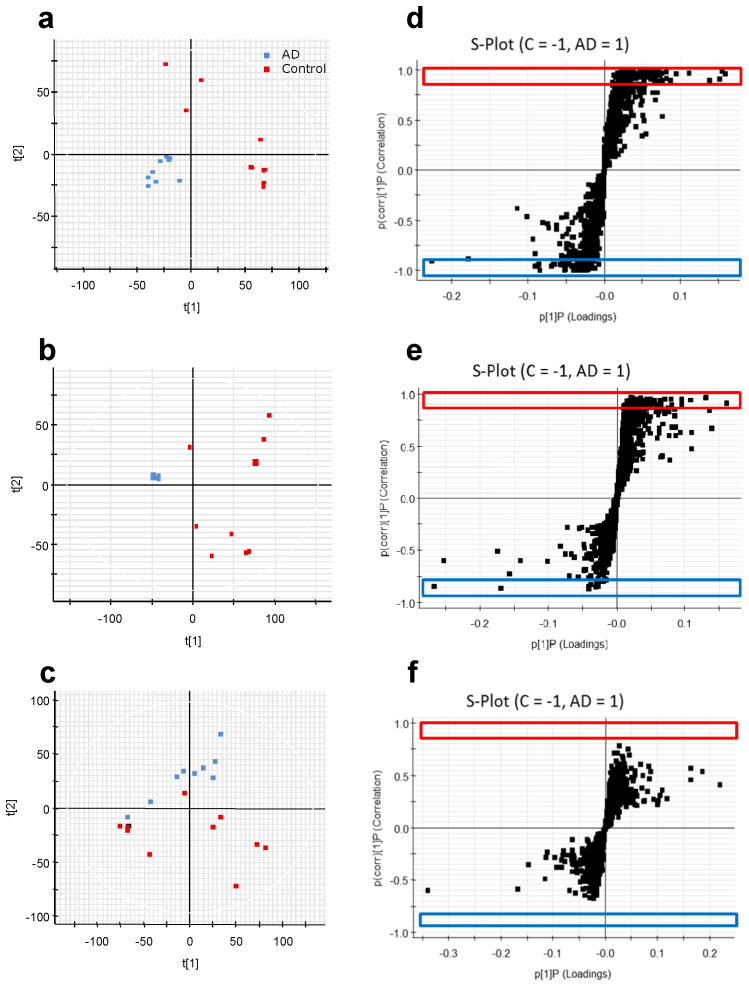
PCA score-plots and S-plots between AD and Control groups in each brain region (n = 10 for each sample). (a), PCA score-plot of frontal lobe region (explained variance (R2): component 1, 0.32 and component 2, 0.49, and predictive ability (Q2): component 1, 0.22 and component 0.21). (b), PCA score-plot of parietal lobe region (R2: component 1, 0.3 and component 2, 0.45, and Q2: component 1, 0.21 and component 2, 0.17). (c), PCA score-plot of occipital lobe region (R2: component 1, 0.18 and component 2, 0.28, and Q2: component 1, 0.08 and component 2, 0.03). (d), S-plot of frontal lobe region. (e), S-plot of parietal lobe region. (f), S-plot of occipital lobe region.

PCA for all samples based on the AD and Control groups revealed an interesting distribution in specific brain regions. The representative PCA score-plots of FL, PL and OL tissues after positive ionization with the T3-C18 UPLC column are shown in Fig. 2a–c. All PCA score-plots are shown in Fig. S2. The FL and PL of the AD group converged on a particular set. On the other hand, the Control groups in all regions indicated a disproportionate lack of coherence in the PCA score-plot patterns of detected *m/z* values. In the OL region, the distributions were rarely different between the AD and Control groups. One feature that these different metabolites share is their contribution to the PCA score-plots with integrated *m/z* values, which indicates that they are very important metabolites for fingerprinting the pathogenesis of AD. Thus, OPLS-DA was used to differentiate between the AD and Control groups in each tissue (n = 10 for each sample) (Fig. 2d–f). Each point represents the metabolite, *m/z* and retention time pair; the X-axis represents variable contribution, and the further this pair point departs from zero, the more the ion contributes to the difference between the two groups of AD and Control; the Y-axis represents variable confidence, and the further the metabolite, *m/z* and retention time pair point departs from zero, the higher the confidence level of the ion is for the difference between the two groups of AD and Control. Hence, in this study, this pair points at the two ends of “S” represent those components contributing most to the difference between the two groups with most confidence, which could be regarded as the most differentiating components between AD and Control. For the extraction of potential markers of group separation, the cut-off of variable confidence was approximately 0.8 for the increased markers and −0.8 for the decreased markers in S-plot^20^,^22^,^23^. Thus, in this study, the S-plot showed that the quality differentiation is employed by the decreased and increased *m/z* values with a correlation of rigorous ± 0.85. In these S-plots, the increased (red-box) and decreased (blue-box) peaks show the important metabolites in the FL and PL regions. This result showed that a total of 431 peaks were analyzed to identify possible biomarker candidates based on all brain regions (Table 2). In the OPLS-DA scores plot (Fig. S3) using Pareto scaling with mean centering, each coordinate represents a brain region, and it could be observed that the determined brain regions are clearly divided into two clusters. All the observations were located within the Hotelling T2 (0.95) ellipse, which confirmed the fact the metabolite difference exists between AD and Control. In addition, univariate analysis was shown in Tables S2 and S3. From the results of the S-plot, specific peaks were extracted from the MS chromatograms of the biomarker candidates in the FL and PL brain regions. We identified five metabolites in both the FL and PL regions based on the shape of the peaks off the T3-C18 column as a common characteristic of all patients, which could be used as possible biomarkers candidates. Based on the available metabolomics databases, a list of retention times, *m/z* values, elemental compositions and possible biomarkers candidates is presented in Table 3. The *m/z* 146.16 and *m/z* 203.22 peaks were speculated to represent SPD and SPM in the AD group. Additionally, these peaks were confirmed by comparison to known standards using UPLC-ESI/TOF/MS chromatograms and spectra (Fig. S4). These results indicated that the *m/z* 146.16 and *m/z* 203.22 peaks were SPD and SPM based on metabolomics databases and standard matching. SPD and SPM are very interesting polyamines with regard to memory-associated brain structures.

### UPLC-ESI/MS/MS analysis of polyamines in brain tissues

**Figure 3:**
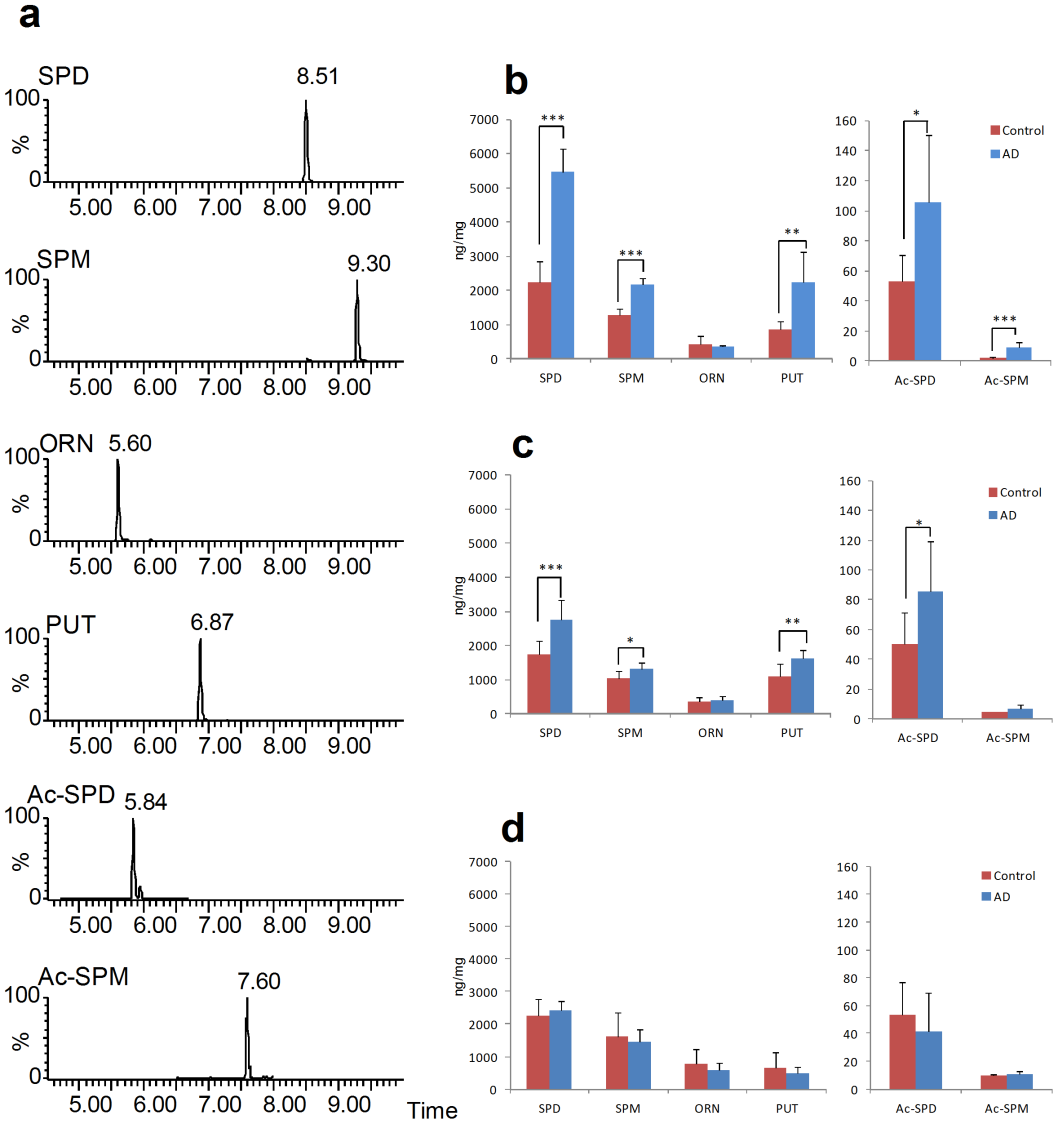
UPLC-ESI/MS/MS with derivatization for the analysis of biogenic polyamine in the brain. (a), SRM chromatograms of polyamine in AD frontal lobe (No. 11). (b), Concentration levels of polyamine in frontal lobe (n = 10 for each sample). (c), Concentration levels of polyamine in occipital lobe (n = 10 for each sample). * P < 0.05, ** P < 0.01, *** P < 0.005.

The analytical determination of biogenic polyamines such as SPD and SPM is not a simple task due to their structure and because they are usually present at low levels in complex matrices. Furthermore, biogenic polyamines do not exhibit a satisfactory absorption at visible, ultraviolet and fluorescent wavelengths. In previous reports, a chemical derivatization was applied for assaying polyamines in biological samples using UPLC-ESI/tandem MS (UPLC-ESI/MS/MS)^24^,^25^. Thus, these polyamines were analyzed using sensitive and selective UPLC-ESI/MS/MS to determine the metabolic mechanism in all brain tissues. UPLC-MS/MS with the derivatization assay was used for the analysis of biogenic polyamines such as SPD, SPM, ornithine (ORN), putrescine (PUT), acetyl-spermidine (Ac-SPD) and acetyl-spermine (Ac-SPM) in brain tissues. Selected reaction monitoring (SRM) chromatograms of the polyamines in the FL region of AD are shown in Fig. 3a. The detection levels of SPD and SPM in the FL and PL regions of the AD samples were increased compared with the OL region, and the difference was statistically significant (Fig. 3b–d). Moreover, PUT, Ac-SPD and Ac-SPM were increased in the FL region of the AD group (Fig. 3b). However, ORN remained at approximately the same levels between the AD and Control groups.

### Metabolite pathway of polyamines

**Figure 4:**
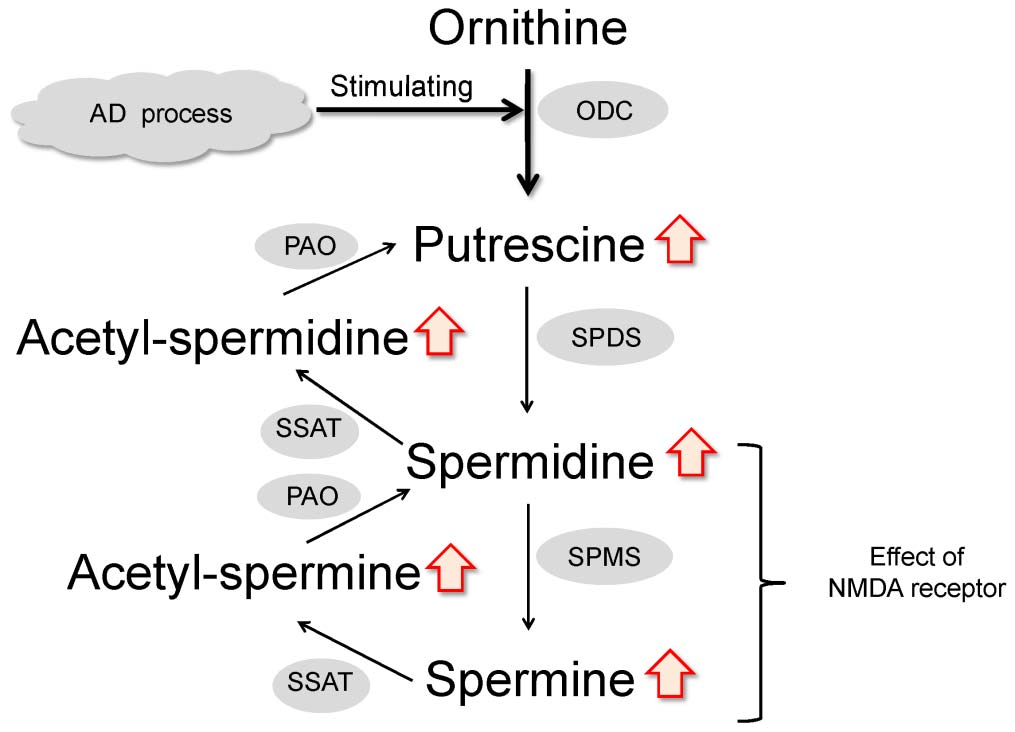
Metabolic pathway of polyamine. Our results indicate increased SPD, SPM, PUT, Ac-SPD and Ac-SPM without a change of ORN in AD pathology. One theory suggests that the NMDA receptor excitotoxicity is caused by an excess of SPD and SPM due to ODC activity induced by plaque and/or tangle deposition in specific brain regions.

To identify the metabolic pathway of SPD and SPM, web-based databases and functional pathway analysis were used for this profiling. The primary pathway was the ornithine decarboxylation system, and the secondary was the SPD and SPM biosynthesis/metabolic pathways (Fig. 4). Location-based metabolite sets for SPD and SPM were found mainly in this system. The detailed construction of the altered polyamine metabolism pathways with a higher score was generated using KEGG as a reference map.

## Discussion

In the present study, we focused on the definitive brain metabolic profiles of low-molecular-weight molecules in specific brain regions with regard to the GB staining properties of plaques/tangles based on samples from reliable AD backgrounds. This novel and accurate UPLC-ESI/TOF/MS of low-molecular-weight molecules allowed for the discovery of the interesting biomarkers SPD and SPM in specific regions of the AD and Control brain tissues. Thus, the use of sensitive and selective UPLC-ESI/MS/MS with a derivatization could allow us to determine the levels of polyamines such as SPD, SPM, ORN, PUT, Ac-SPD and Ac-SPM, in specific brain regions related to AD pathology. Based on our data, the polyamine metabolite pathway in a specific brain region is likely to be responsible for up-regulated polyamine metabolism and elevated ornithine decarboxylase (ODC) activity (Fig. 4). In addition, our results support the hypothesis that *N*-methyl-*D*-aspartate (NMDA) receptor excitotoxicity is caused by excess SPD and SPM, and that ODC activity was stimulated by the effects of AD processes in localized brain regions. Our approach overcomes the limitations of previous pathological examinations using antibody-targeted analytes and stained plaques and tangles in biological samples from AD patients.

Metabolomics has substantially advanced our understanding of the analytical features that constitute part of AD research^26^,^27^,^28^,^29^. Several studies have propsed or performed metabolic profiling of low-molecular-weight molecules in biological AD samples, such as CSF and plasma based on MS techniques^15^,^16^,^17^,^18^,^19^,^30^,^31^. In these previous studies, potential biomarkers have revealed insights into the presence of sphingomyelin, ceramide and desmosterol in AD plasma^15^,^31^. A peripheral plasma biomarker would offer significant advantages over these approaches, allowing acceptable clinical screening and monitoring of the AD state. While it is known that there is communication between peripheral and central pathology based on data from these reliable backgrounds, the utility of plasma biomarkers measurements has remained limited. Pathological and diagnostic issues likely contribute to these limitations, thereby underlining the need for a better understanding of the dynamics of metabolites for use in diagnosis as well as the need for thorough studies to determine the changes in low-molecular-weight molecules in specific brain regions with regard to plaques/tangles. Thus, definitive brain metabolomics is a powerful and distinct procedure that can be applied to the discovery of biomarkers of AD pathology. These are a few reports that have investigated the metabolome of the AD brain via MS techniques^12^,^32^,^33^,^34^. Among them, Graham et al. investigated the human brain metabolome to identify potential low-molecular-weight molecules for AD using an UPLC-ESI/QTOF/TOF/MS^32^. Unfortunately, they did not identify any reasonable low-molecular-weight molecules, which indicates that determining the pathology of AD based on plaques/tangles and that obtaining precious information of brain tissues, such as region, pathological examination, and genetic code of ApoE, is difficult^32^. In this experiment, samples with a genetic genotype of ApoE 3/3 were taken from the Fukushimura Brain Bank for metabolic profiling. ApoE is a major cholesterol carrier that supports lipid transport and injury repair in the brain. The presence of the ApoE 4 allele is also associated with an increased risk of cerebral AD pathology and age-related cognitive decline during normal aging^35^,^36^,^37^. Thus, it is necessary to consider the relationship between genetic risk factors and markers for AD pathology for future metabolomics, proteomics and imaging studies^38^. Moreover, many researchers have discussed evidence for underlying AD mechanisms, including an important relationship between resting state functional connectivity and the resulting distinctive pattern of Aβ plaque deposition in specific brain regions^39^,^40^,^41^,^42^. Thus, we should be able to predict the changes of metabolism in specific regions regarding the deposition of Aβ plaques. Based on an accurate experimental and pathological background, we identified increased SPD and SPM in AD brains by brain metabolomics studies in all patients.

Endogenous polyamines such as SPD and SPM have the ability to modulate various ion channels in the brain specifically related to the NMDA receptor^43^,^44^,^45^,^46^. The NMDA receptor antagonist memantine is the first in a novel class of AD medications that act on the glutamatergic system, and it has been shown to have a modest effect in moderate-to-severe disease^47^. Dysfunction of the NMDA receptor, manifested as neuronal excitotoxicity, is hypothesized to be involved in the etiology of AD^48^. The balance between these mechanisms within specific neuronal networks may determine whether the activity of the NMDA receptor increases or decreases Aβ *in vivo*^49^. Aβ pathology may drive an abnormal conformation of the NMDA receptor or deleteriously enhance the association of the NMDA receptor with certain molecules^50^. For example, the NMDA subunit could be stimulated by SPM in the dimeric pairs of NR1 and NR2, and NR1 and NR2A^51^. The specific polyamine that binds to the NMDA receptor may contribute more to local variability in brain injury than the glutamate site, and it has the potential to ameliorate excitotoxicity in specific AD-related brain regions^52^. Thus, our discovery of an abnormal increase in the localized concentration of SPD and SPM indicated that over-excitation mediated by specific NMDA receptors might contribute to localized focal brain in AD.

The effects of abnormal increases in the concentration of neural polyamines are not clear. In this discussion, we focus on the apparent cause-and-effect chain of abnormal elevated polyamines in the AD brain region. For example, Aβ peptides have been shown to be responsible for up-regulated polyamine metabolism, specifically, increased polyamine uptake and elevated ODC activity^53^. In addition, Yatin et al. reported that Aβ causes an increase in polyamine metabolism manifested by up-regulated SPM uptake and increased ODC activity and that up-regulation of polyamine metabolism due to Aβ is a result of enhanced neurotoxicity^54^. These extraordinary behaviors of Aβ plaque deposition, polyamine metabolism, ODC activity and NMDA receptor excitotoxicity occurred in localized AD brain regions^55^. Fig. 4 shows the metabolism of polyamines from a web-based database and functional pathway analysis. PUT is synthesized from ORN by ODC. SPD is then synthesized from PUT, and SPM from SPD, by transfer of the aminopropyl moiety of decarboxylated *S*-adenosylmethionine. The enzymes catalyzing these reactions are spermidine synthase (SPDS) and spermine synthase (SPMS). Different enzymes convert SPD to SPM, and SPM to PUT. Spermine oxidase (SMO) directly catalyzes the conversion of SPM to SPD, and both spermidine/spermine *N*-acetyltransferase (SSAT) and acetylpolyamine oxidase (PAO) catalyze the conversion of Ac-SPM to SPD and Ac-APD to PUT. To investigate the regulation of the polyamine content, the concentrations of SPD, SPM, ORN, PUT, Ac-SPD and Ac-SPM in brain tissues were analyzed by UPLC-ESI/MS/MS. In previous reports, a chemical derivatization was applied for assaying polyamines in biological samples using sensitive and selective UPLC-ESI/MS/MS to determine the metabolic mechanism. UPLC-ESI/MS/MS with the derivatization assay was used for the analysis of biogenic polyamines such as SPD, SPM, ORN, PUT, Ac-SPD and Ac-SPM in brain tissues. SRM chromatograms of the polyamines are shown in Fig. 3a. Our results indicate that increased SPD, SPM, PUT, Ac-SPD and Ac-SPM without a change of ORN are associated with ODC activity in the AD processes (Fig. 4). Thus, one theory posits that the state-dependency effect of excess SPD and SPM in focal brain would be adverse impact of NMDA receptor.

## Methods

### Materials

Polyamine standards such as SPD, SPM, ORN, PUT, Ac-SPD and Ac-SPM were purchased from Wako Pure Chemical Co. (Osaka, Japan), Kanto Chemical Co. (Tokyo, Japan) and Sigma-Aldrich Co. (St. Louis, MO). 1,6-Diaminohexan (DAH) and 4-(*N*,*N*Dimethylaminosulfonyl)-7-fluoro-2,1,3-benzoxadiazole (DBD-F) were obtained from Tokyo Chemical Industry Co. (Tokyo, Japan). DAH was used as the internal standard. Organic solvents, sodium tetraborate (Borax) and 1 mol/L Tris-HCl (pH 7.5) were purchased from Wako Pure Chemical Co. (Osaka, Japan). Formic acid (FA), methanol and acetonitrile of LC-MS grade were obtained from Kanto Chemical Co. (Tokyo, Japan). All other chemicals were of analytical grade and were used without further purification. De-ionized and distilled water was used throughout the study (Aquarius PWU200 automatic water distillation apparatus, Advantec, Tokyo, Japan).

### Autopsy and sampling of brain tissues

The utilized tissues from the Fukushimura Brain Bank were used for the accurate, reliable and detailed pathological evaluation of AD^56^. The tissues samples were confined to specific brain regions, were of the ApoE 3/3 gene type, had visual pathology of plaques/tangles, and the patients had been diagnosed with gradual pre-mortem memory loss. The ApoE genotype affects Aβ accumulation in the pathogenesis of AD, cognition and functional variation^57^,^58^,^59^. We obtained written informed consent for autopsy from the patients' guardians as well as their permission to use the results of diagnosis, research, and genetic analysis at the Choju Medical Institute Fukushimura Hospital. The brain was removed at autopsy, weighed, cut midsagittally and examined for vascular and other macroscopically detectable lesions. Specimens for diagnostic examination were taken from the hemisphere that showed abnormalities on CT scanning or from the left hemisphere if no difference between the left and the right hemisphere was found by CT. Specimens were separated into the cerebrum, cerebellum, midbrain, pons and medulla and dissected into slices. These slices were fixed in buffered 10% formalin as a hemispheric block. The other brain regions were also separated, and stored at −80°C until use and used after gaining approval from authorities including the University of Shizuoka ethics panel.

GB staining was carried out to screen for senile plaques and neurofibrillary tangles in several brain regions^60^,^61^. For routine immunohistochemistry, an anti-human β amyloid antibody (Dako, mouse monoclonal, 1∶30) was used for detecting senile plaques, an anti-human pTau antibody (INNOGENETICS, clone AT8 1∶1000) for detecting tangles, and an anti-phosphorylated α-synuclein antibody (Wako, mouse monoclonal clone pSyn#64 1∶1000) for detecting Lewy bodies and neuritis.

All of our cases were reviewed and discussed with several doctors at a clinicopathological conference^62^. Senile plaques were confirmed using criteria of the Consortium to Establish a Registry for Alzheimer's Disease (CERAD) and Braak and Braak staging, and a grade higher than C was selected as indicative of AD^63^,^64^. The ApoE genotype was analyzed and correlated with neuropathological complications. DNA was extracted from brain tissues by the phenol-chloroform method. Peripheral blood of the elderly in the PBC group was collected in tubes containing EDTA, and DNA was extracted using a QIAamp DNA Blood kit (Qiagen, Valencia, CA) and stored at 4°C. ApoE genotyping was carried out by the polymerase chain reaction (PCR)-restriction fragment length polymorphism (RFLP) method^65^.

### UPLC-ESI/TOF/MS analysis for metabolic profiling

The UPLC system was a Waters Acquity H Class (Waters Co., Milford, MA). The reversed phase analysis was performed using an Acquity UPLC HSS T3 C18 column (1.8 μm, 2.1 × 100 mm) [T3-C18] and Discovery HS-F5 HPLC Column (3 μm, 150 mm × 2.1 mm, SUPELCO) [HS-F5] at 40°C. The injection volume was 5 μL. The mobile phase consisted of solvent A, 0.1% FA in water, and solvent B, 0.1% FA in acetonitrile, was delivered at a flow rate of 0.4 mL/min for T3-C18 or 0.3 mL/min for HS-F5. This mobile phase used in gradient mode for the simple separation of low-molecules in two columns. The gradient elution was as follows: T3-C18; B% = 2-2-98-98 (0-3-11-12 min), and HS-F5; B% = 0-0-100-100 (0-10-25-30 min), respectively. The separated compounds were detected by a Waters LCT Premier XE time-of-flight mass spectrometer (TOF/MS) (Waters Co., Milford, MA). The positive and negative electrospray (ESI) ionization mode conditions were as follows: capillary voltage was 3.0 kV, sample cone was 15 V, source temperature of 120°C and desolvation temperature of 350°C. The cone and desolvation gas flows were 50 and 650 L/h, respectively, and were obtained using a nitrogen source. The analytical mode and dynamic range were the V mode and normal. The aperture 1 voltage was 15 V. For calibration, the reference solution used 4 μg/mL leucine enkephalin (*m/z* 556.28) in 0.1% FA in water/acetonitrile (50/50, V/V). The scan mode used was from *m/z* 100 to 1000.

### Sample preparation of low-molecular-weight molecules in brain tissues

These supernatant solutions were applied to centrifugal ultrafiltration using Amicon Ultra-0.5 mL 3 K (Ultracel-3K, regenerated cellulose 3,000 M.W., Millipore Co. Ltd., Billerica, MA). In the first step, the brain tissues and two zirconia beads (5.0 mm) in the tubes were placed in the holes of an aluminum block and immediately homogenized for 3 min by a Shake Master (Bio Medical Sciences, Tokyo, Japan). In the second step, the homogenized tissues were weighed in a tube, and 500 μL of the extraction solvent, i.e., the mixture of methanol and Tris-HCl buffer (50 mM, pH 7.4)/NaCl (200 mM)/EDTA (2 mM) in water [1∶1], was added to 50 mg tissue (at the ratio of 100 mg/mL). For the third step, the extraction solution and two zirconia beads (3.0 mm) in the tubes were homogenized for 3 min by a Shake Master again. Finally, these supernatant solutions were applied to centrifugal ultrafiltration using Amicon Ultra-0.5 mL 3 K. A 5 μL aliquot of the filtrate was then subjected to the UPLC-ESI/TOF/MS system.

### Validation of UPLC-ESI/TOF/MS system

The quality control in the pooled parietal lobe tissues from AD (n = 10) and Control (n = 10) were performed on the same day for determining the intra-day accuracy, replicate (n = 6) analytes of various peaks. The intra-day accuracy was expressed as the relative standard deviation (RSD, %) of retention time and peak area for typical *m/z* values (. For multivariate data analysis, each sample of real patients was obtained from single running based on this validation.

### Multivariate data analysis of *m/z* values

The UPLC-ESI/TOF/MS data were analyzed for peak detection and alignment from *m/z* 100 to 1000, and exported for PCA and OPLS-DA by MarkerLynxTM XS V4.1 SCN803 (Waters Co., Milford, MA). The method parameters were as follows: mass tolerance = 0.05 Da, apex track peak parameters, peak width at 5% height (seconds) = 15/peak-to-peak baseline noise = 50, apply smoothing = yes, collection parameters, intensity threshold (counts) = 100/mass window = 0.05/retention time window = 0.10, noise elimination level = 6, deisotope data = yes. R2 (cumulative) and Q2 (cumulative) were used to determine the validity of the model. R2 (cum) indicates the variation described by all components in the model, and Q2 is a measure of how accurately the model can predict class membership. In univariate analysis of metabolites, the decreased and increased *m/z* values with a correlation of rigorous ± 0.85 were extracted by the estimation of peak shape and complete separation in extracted ion monitoring. Then, these averages of peak area from all sample of AD and Control showed a significant differences in Tables S2 and S3.

### Identification of low-molecular-weight molecules by database comparison

The elemental compositions of the low-molecular-weight molecules on the S-plot were identified based on the mass and retention time, and the values of mDa (the difference from the exact mass) and i-FIT (the correctness of isotope patterns of elemental composition; the lower i-FIT normalized values mean high) of each compound. Low-molecular-weight molecules with good mDa values and i-FIT levels were extracted from these prospective formulas and the MS spectra of the unknowns were matched to standard model compounds. The MarkerLynxTM XS V4.1 SCN803 combined lists of biomarkers candidates extracted from the following five metabolomics databases: NIST (http://www.nist.gov/pml/data/asd.cfm), MassBank (http://www.massbank.jp/), KEGG (http://www.kegg.com/), BioCyc (http://biocyc.org/), and the human metabolite database (http://www.hmdb.ca/). A mass tolerance of 5.0 mDa was set as well as a maximum elemental composition of C = 500, H = 1000, N = 200, O = 200, S = 10, P = 10, and Cl = 10. The software automatically filters compounds from different libraries that have the same KEGG.

### Analysis of polyamine in brain tissues

A UPLC-ESI/MS/MS was used for measuring polyamines as derivatives in brain tissues. A 20-μL extraction solution of brain tissues using the above protocol was added to 20 μL of acetonitrile containing the internal standard (DHA 500 nmol/L). Then, the mix solution was dried using a Personal Evaporator (EZ-2, Genevac Co., NY). The residues was added to 150 μL of DBD-F in acetonitrile (40 mmol/L) and 150 μL of 100 mmol/L Borax (pH 9.3) at 60°C for 30 min, and then UPLC-ESI/MS/MS was performed. The UPLC system used a Waters ACQUITY UPLC (Waters, Milford, MA, USA). The UPLC separation was achieved using a Waters Acquity UPLC BEH C18 (2.1 × 100 mm, 1.7 μm; Waters, Milford, MA) maintained at 40°C and the mobile phase consisted of 0.1% FA in water (Solvent A) and 0.1% FA in acetonitrile (Solvent B). The gradient was as follows: B% = 20-60-90 (0-8-10 min), with a flow rate of 0.4 mL/min. The injection volume was 5 μL. The separated compounds were detected using a Waters Xevo™ TQ-S triple quadrupole mass spectrometer (Waters, Milford, MA). The mass spectrometer was operated with an electrospray source in the positive ionization mode. The ESI source conditions were a capillary voltage of 3.0 kV, source offset of 50 V and desolvation temperature of 500°C. The cone and desolvation gas flow rates were 150 L/hr and 1000 L/hr, respectively and were obtained from a nitrogen source (N_2_ Supplier Model 24S, Anest Iwata Co., Yokohama, Japan). Argon was used as the collision gas and was regulated at 0.15 mL/min. The LH resolution 1, HM resolution 1, ion energy 1, LM resolution 2, HM resolution 2, and ion energy 2 were 3.0, 15.0, 0.5, 3.0, 15.0 and 1.0, respectively. The separation, detection conditions and monitoring ions are the same as those listed in Table S4.

## Author Contributions

K.I. and H.T. carried out most of the metabolomics experiments. H.A. and Y.H. performed the pathological examination, and H.A. designed and supervised the clinical work for the AD patients. K.I. built the basic concept and contributed to the manuscript preparation. N.M. conducted metabolite pathway investigation. K.I., H.T., H.A., T.Y. and T.T. contributed to preparation of materials and provided advice on project planning and data interpretation. K.I. and H.A. designed and supervised the project, and wrote the manuscript.

## Supplementary Material

Supplementary Information Supplementary Information

## Figures and Tables

**Table 1:** Characterization of the AD and Control study groups

No.	Groups	Age/Sex [Onset age]	Pathological diagnosis [Case of death]	Brain fix (hr)	Brain weight [g]
1	Control	85/F [None]	MI [Ileus]	4	1095
2	Control	95/F [85]	MI (Respiratory failure)	24	990
3	Control	87/F [86]	Multiple myeloma (Multiple myeloma)	10	1060
4	Control	95/F [None]	MI (Bile duct cancer)	12	1190
5	Control	82/M [None]	Subdural hemorrhage (Respiratory failure)	14	1320
6	Control	79/M [None]	MI (Heart failure)	13	1080
7	Control	95/F [85]	MI (Heart failure)	4	870
8	Control	87/F [None]	Cerebellum infarct (Unknown)	36	1080
9	Control	90/M [None]	Traumatic Subarachnoid (Trauma)	4	1190
10	Control	93/F [None]	Physiological aging (Heart failure)	2	950
11	AD	87/F [81]	AD (Braak VI/C), CAA [Anemia)	6.5	960
12	AD	95/F [86]	AD (Braak VI/C) (Heart failure)	6	920
13	AD	91/F [76]	AD (Braak VI/C), MI (Respiratory failure)	3.5	980
14	AD	93/F [86]	AD (Braak VI/C), CAA (Heart failure)	3	1180
15	AD	86/M [80]	AD (Braak VI/C) (Pneumonia)	12	990
16	AD	73/F [53]	AD (Braak VI/C) (Heart and respiratory failure)	2.5	710
17	AD	97/M [91]	AD (Braak V/C), MI (Multi organ failure)	12	1160
18	AD	88/F [82]	AD (Braak V/C), MI (Pneumonia and heart failure)	11	1010
19	AD	91/M [84]	AD (Braak V/C) (Respiratory failure)	5	1260
20	AD	91/F [81]	AD (Braak V/C), MI (Sudden death)	20	1000

*Bottom note*: M: male, F: female, MI: multiple infarct, CAA: cerebral amyloid angiopathy.

**Table 2:** A total of 431 peaks representing possible biomarker candidates based on all brain regions

Ionization	Positive mode				Negative mode			
	T3-C18		HS-F5		T3-C18		HS-F5	
Column	Increase	Decrease	Increase	Decrease	Increase	Decrease	Increase	Decrease
FL	65	62	57	33	21	28	9	5
PL	56	11	32	16	9	0	0	1
OL	0	0	16	3	0	3	1	3

**Table 3:** Possible biomarkers candidates for AD from increased *m/z* values based on the metabolomics databases

Brain region	Possible biomarkers	Ret. Time	*m/z*	Elemental Composition (mDa, i-FIT)
FL	Spermidine	0.6022	146.1656	−0.1, 0.0, C_7_H_20_N_3_
PL	Spermidine	0.6046	146.1658	0.1, 0.0, C_7_H_20_N_3_
FL	Spermine	0.618	203.2221	−1.5, 0.0, C_10_H_27_N_4_
PL	Spermine	0.5938	203.2226	−1.0, 0.0, C_10_H_27_N_4_
FL	3-{(R)-(Dimethylamino)[(1R,2R)-2-hydroxycyclohexyl]methyl}phenol	8.5688	250.1775	−3.2, 0.8, C_15_H_24_NO_2_
PL	3-{(R)-(Dimethylamino)[(1R,2R)-2-hydroxycyclohexyl]methyl}phenol	8.202	250.1796	−1.1, 1.8, C_15_H_24_NO_2_
FL	Unknown	10.8745	343.2235	2.9, 1.3, C_22_H_31_O_3_
PL		10.4778	343.2286	1.3, 3.5, C_22_H_31_O_3_
FL	Unknown	5.5884	416.2824	3.0, 1.4, C_17_H_42_N_3_O_6_S
PL		5.8498	416.284	−0.7, 0.8, C_17_H_42_N_3_O_6_S

*Bottom note*: Both FL and PL regions based on the shape of each peak using T3-C18 column as a common characteristic of all patients.
