# Supplementary material for: Metabolic profiling of Alzheimer's disease brains
Source: Sci Rep. 2013 Aug 6;3:2364. doi: 10.1038/srep02364 (PMC3734482; doi:10.1038/srep02364)
Supplement: Supplementary Information [file srep02364-s1.doc]

**Supplementary Information**

**Metabolic profiling of Alzheimer’s disease brains**

Koichi Inoue^1*^, Haruhito Tsutsui^1^, Hiroyasu Akatsu^2, 3^, Yoshio Hashizume^2^, Noriyuki Matsukawa^3^, Takayuki Yamamoto^2^ & Toshimasa Toyo’oka^1*^

1: Laboratory of Analytical and Bio-Analytical Chemistry, School of Pharmaceutical Sciences, University of Shizuoka, Shizuoka, Japan

2: Department of Neuropathology, Choju Medical Institute, Fukushimura Hospital, Toyohashi, Japan

3: Department of Neurology, Nagoya City University, Graduate School of Medical Sciences, Nagoya, Japan

* Correspondence and requests for materials should be addressed to K.I. (kinoue@u-shizuoka-ken.ac.jp) or T.T. (toyooka@u-shizuoka-ken.ac.jp)

**Figure S1**: UPLC-ESI/TOF/MS chromatograms (T3-C18 and positive ionization) and list of low-molecules in brain tissues from AD and Control groups.

a, The chromatogram of low-molecules in FC brain from control

b, The chromatogram of low-molecules in FC brain from AD

c, The list of detected low-molecules in brain tissues

**Figure S2**: PCA score-plots between AD and Control groups in each brain region.

a, PCA score-plot of FL region on positive ionization with HS-F5 UPLC column (R2: component 1, 0.27 and component 2, 0.45, and Q2: component 1, 0.16 and component 2, 0.23)

b, PCA score-plot of PL region on positve ionization with HS-F5 UPLC column (R2: component 1, 0.16 and component 2, 0.30, and Q2: component 1, 0.02 and component 2, 0.02)

c, PCA score-plot of OL region on positive ionization with HS-F5 UPLC column (R2: component 1, 0.20 and component 2, 0.33, and Q2: component 1, 0.03 and component 2, -0.06)

d, PCA score-plot of FL region on negative ionization with T3-C18 UPLC column (R2: component 1, 0.34 and component 2, 0.55, and Q2: component 1, 0.23 and component 2, 0.40)

e, PCA score-plot of PL region on negative ionization with T3-C18 UPLC column (R2: component 1, 0.27 and component 2, 0.45, and Q2: component 1, 0.06 and component 2, -0.02)

f, PCA score-plot of OL region on negative ionization with T3-C18 UPLC column (R2: component 1, 0.21 and component 2, 0.31, and Q2: component 1, 0.05 and component 2, -0.05)

g, PCA score-plot of FL region on negative ionization with HS-F5 UPLC column (R2: component 1, 0.25 and component 2, 0.41, and Q2: component 1, 0.02 and component 2, 0.02)

h, PCA score-plot of PL region on negative ionization with HS-F5 UPLC column (R2: component 1, 0.30 and component 2, 0.50, and Q2: component 1, 0.11 and component 2, 0.21)

i, PCA score-plot of OL region on negative ionization with HS-F5 UPLC column (R2: component 1, 0.19 and component 2, 0.37, and Q2: component 1, -0.04 and component 2, -0.09)

**Figure S3**: OPLS-DA score-plots between AD and Control groups in each brain region.

a, OPLS-DA score-plot of FL region on positive ionization with T3-C18 UPLC column

b, OPLS-DA score-plot of PL region on positive ionization with T3-C18 UPLC column

c, OPLS-DA score-plot of OL region on positive ionization with T3-C18 UPLC column

**Figure S4**: UPLC-ESI/TOF/MS chromatograms and spectra of SPD and SPM standard and brain sample.

a, The chromatogram(extracted ion: m/z 146.16) and mass spectrum of SPD standard on positive mode.

b, The chromatogram chromatogram (extracted ion: m/z 146.16) and mass spectrum of SPD in brain sample (FL region of AD).

c, The chromatogram (extracted ion: m/z 203.22) and mass spectrum of SPM standard on positive mode.

d, The chromatogram (extracted ion: m/z 203.22) and mass spectrum of SPM in brain sample (FL region of AD).

**Table S1.** Validation of UPLC-ESI/TOF/MS analysis of typical *m/z* values in pooled PL brain tissues from AD and Control.

*The pooled parietal lobe tissues from AD (n=10) and Control (n=10) were performed on the same day for determining the intra-day accuracy, replicate (n=6) analytes of various peaks.

**Table S2.** Univariate analysis of metabolites from UPLC-ESI/TOF/MS data based on S-plot in FL brain.

**Table S3.** Univariate analysis of metabolites from UPLC-ESI/TOF/MS data based on S-plot in PL brain.

**Table S4.** The SRM conditions for the analysis of polyamines in brain tissues.
